# Visual mismatch negativity (vMMN): a prediction error signal in the visual modality

**DOI:** 10.3389/fnhum.2014.01074

**Published:** 2015-01-22

**Authors:** Gábor Stefanics, Piia Astikainen, István Czigler

**Affiliations:** ^1^Translational Neuromodeling Unit, Institute for Biomedical Engineering, University of Zurich and ETH ZürichZurich, Switzerland; ^2^Laboratory for Social and Neural Systems Research, Department of Economics, University of ZurichZurich, Switzerland; ^3^Department of Psychology, University of JyväskyläJyväskylä, Finland; ^4^Research Center for Natural Sciences, Institute of Cognitive Neuroscience and Psychology, Hungarian Academy of SciencesBudapest, Hungary; ^5^Department of Cognitive Psychology, Institute of Psychology, Eötvös Loránd UniversityBudapest, Hungary

**Keywords:** EEG, ERP, perceptual learning, predictive coding, prediction error, repetition suppression, stimulus specific adaptation, visual mismatch negativity

Our visual field contains much more information at every moment than we can attend and consciously process. How is the multitude of unattended events processed in the brain and selected for the further attentive evaluation? Current theories of visual change detection emphasize the importance of conscious attention to detect changes in the visual environment. However, an increasing body of studies shows that the human brain is capable of detecting even small visual changes if such changes violate non-conscious probabilistic expectations based on prior experiences. In other words, our brain automatically represents environmental statistical regularities.

Since the discovery of the auditory mismatch negativity (MMN) event-related potential (ERP) component, the majority of research in the field has focused on auditory deviance detection. Such automatic change detection mechanisms operate in the visual modality too, as indicated by the visual mismatch negativity (vMMN) brain potential to rare changes. vMMN is typically elicited by stimuli with infrequent (deviant) features embedded in a stream of frequent (standard) stimuli, outside the focus of attention. Information about both simple and more complex characteristics of stimuli is rapidly processed and stored by the brain in the absence of conscious attention.

In this research topic we aim to present vMMN as a prediction error signal and put it in context of the hierarchical predictive coding framework. Predictive coding theories account for phenomena such as MMN and repetition suppression, and place them in a broader context of a general theory of cortical responses (Friston, 2005, 2010). Each paper in this Research Topic is a valuable contribution to the field of automatic visual change detection and deepens our understanding of the short term plasticity underlying predictive processes of visual perceptual learning.

A wide range of vMMN studies has been presented in seventeen articles in this Research Topic. Twelve articles address roughly four general sub-themes including attention, language, face processing, and psychiatric disorders. Additionally, four articles focused on particular subjects such as the oblique effect, object formation, and development and time-frequency analysis of vMMN. Furthermore, a review paper presented vMMN in a hierarchical predictive coding framework.

Four articles investigated the relationship between attention and vMMN. Kremláček et al. (2013) presented subjects with radial motion stimuli in the periphery of the visual field using an oddball paradigm and manipulated the attentional load by varying the difficulty of a central distractor tasks. They aimed to manipulate the amount of available attentional resources that might have been involuntarily captured by the vMMN-evoking stimuli presented in the periphery outside of the attentional focus. The distractor task had three difficulty levels: (1) a central fixation (easy), and a target number detection task with (2) one target number (moderate), and (3) three target numbers (difficult). Analysis of deviant minus standard differential waveforms revealed a significant posterior negativity in the ~140–200 ms interval, which was unaffected by the difficulty of the central task, indicating that the automatic processes underlying registration of changes in motion are independent of attentional resources used to detect target numbers.

Kimura and Takeda (2013) investigated whether characteristics of vMMN depended on the difficulty of an attended primary task, i.e., they tested the level of automaticity of the vMMN. Task difficulty was manipulated as the magnitude of change of a circle at fixation, and vMMN was elicited by deviant orientation of bar patterns. An equal probability control condition was also used. The difference potential between the deviant-related ERP and the ERP elicited by identical orientation pattern in the control condition appeared to be influenced by the difficulty of the attentive task. As a function of task difficulty, the latency of the difference potential (i.e., the vMMN) increased, indicating that processes underlying vMMN to orientation changes are not fully independent of the attention demands of the ongoing tasks.

Kuldkepp et al. (2013) used rare changes in direction of peripheral motion to evoke vMMN applying a novel continuous whole-display stimulus configuration. The demanding distractor task involved motion onset detection and was presented in the center of the visual field. The level of attention to the vMMN-evoking stimuli was varied by manipulating their task-relevance using “Ignore” and “Attend” conditions. Deviant minus standard waveforms in the “Ignore” condition showed significant vMMN in the 100–200, 250–300, and 235–375 ms intervals, whereas in the “Attend” condition only in the later 250–400 ms interval a reliable vMMN was observed, indicating that task-relevance eliminates the difference observable in early components to task irrelevant deviant and standard stimuli.

van Rhijn et al. (2013) used a binocular rivalry situation to investigate whether vMMN is generated by low levels of the visual system at which simple features are processed, or by higher levels. Attention to the vMMN-evoking stimuli was manipulated by their task-relevance, in the “reduced-attention” condition participants performed a two-back task presented at fixation, whereas in the “attend-to-rivalry” they recorded their experiences of rivalry by button presses. Oddball series emerged only if the stimuli from the two eyes were treated separately, i.e., combining the stimuli from the eyes produced no deviant-standard separation, but two equiprobable orientations of a grating pattern. VMMN emerged in the 130–160 and 196–226 ms intervals both when the stimulus stream was task-irrelevant and when it was attended. The results indicate that vMMN may emerge in visual structures before the level of binocular integration.

Two studies in this Topic demonstrate the potential of vMMN in studying language-related phenomena. Shtyrov et al. (2013) used vMMN to investigate early automatic lexical effects in the visual modality. They presented participants with word and pseudo-word stimuli perifoveally using an oddball design, while participants engaged in a centrally presented task. Significant vMMN responses were observed to words at the 100–120 and 240-260 ms latency ranges and the authors concluded that early processing of orthographic stimuli can take place automatically outside the focus of attention.

Files et al. (2013) presented consonant-vowel syllables visually using videos of talking faces. Within the oddball sequences task-irrelevant deviant and standard syllables were presented, as well as task-relevant target syllables. The syllables were phonetically near (e.g., “zha” vs. “ta”), or far (e.g., “zha” vs “fa”). The main interest of the study was the lateralization of the vMMN. In the left posterior temporal areas area only the phonetically far deviant elicited vMMN. However, sound difference *per-se* elicited vMMN in the right temporal areas, independent of the phonetic distance. The results also show the influence of speech-related processing on visual change detection.

The auditory MMN has proved extremely useful in studying cognitive deficits in neuropsychiatric and neurological diseases. There is hope that studies using visual MMN can further our understanding of a variety of disorders, too. Three studies in this Topic used vMMN to investigate clinically relevant issues. Maekawa et al. (2013) applied a three-stimulus oddball paradigm in patients with bipolar disorder. Rare changes in spatial frequency of windmill pattern stimuli were used to elicit vMMN while subjects performed a task which involved detection of rare white discs (target) and simultaneously listened to an acoustically presented story. The vMMN component was smaller in patients than in control participants, indicating impairment in automatic visual predictive mechanisms in bipolar disorder.

Cléry et al. (2013) investigated predictive visual processing in patients with autism spectrum disorder (ASD). Adult ASD patients and control participants were compared in a three-stimulus passive oddball paradigm. The participants' task was to detect the disappearance of the fixation point. The standard or deviant stimuli were frequent horizontal and rare vertical deformations of a circle into an ellipse, respectively, while deformation into another shape served as a novel stimulus. Deviant deformation elicited smaller vMMN in the patient group. In the control group a subsequent ERP component, the orientation-related P3a, emerged only to the novel stimuli. However, in the ASD group the deviant stimulus also elicited the P3a, indicating altered change-detection and orientation processes in the ASD group.

Four studies used vMMN to investigate face processing. Gayle et al. (2012) investigated whether vMMN evoked by rare changes in unattended facial expressions can be used to predict autism spectrum personality traits as measured by the Adult Autism Spectrum Quotient in healthy adults. Emotionally neutral faces served as frequent standard stimuli, whereas rare happy and sad faces served as deviant stimuli in an oddball paradigm while participants engaged in a separate task. Deviant emotions elicited a posterior vMMN response at 150–425 ms, which correlated with the autism quotient. The authors concluded that vMMN might be useful as an objective index of affective reactivity in ASD.

Detection of changes in facial expressions was also explored by Astikainen et al. (2013). ERPs were recorded to pictures of neutral, fearful, and happy faces using oddball and equiprobable stimulus conditions presenting rare emotional faces among neutral ones or all three expressions with equal probability, respectively. Independent component analysis applied to the emotional minus neutral differential responses revealed two prominent components in both stimulus conditions in the 100–200 s interval. A component peaking at 130 ms showed a difference in scalp topography between oddball and equiprobable conditions. This bilateral component at 130 ms in the oddball condition conformed to vMMN. Moreover, it was distinct from face sensitive N170 which was modulated by the emotional expression only. Results suggest that future vMMN studies should take into account possible confounding effects caused by the differential processing of the emotional expressions as such.

Kreegipuu et al. (2013) presented participants with schematic faces of neutral, happy and angry expressions while they were attending to scrambled faces presented in the same series. Two stimulus presentation conditions were compared, an oddball and an optimum paradigm, the latter involving several different deviant facial emotions. VMMN was elicited similarly in both conditions at posterior sites. Angry deviant faces elicited larger vMMN responses than happy deviant faces irrespectively of the paradigm type. The results encourage using a multi-feature “optimum” paradigm to study predictive processes related to different facial emotions.

Processing of the gender information from the faces was investigated by Kecskés-Kovács et al. (2013). Female and male faces were applied as standard and deviant stimuli using an oddball design with two different stimulus-onset asynchronies. Faces with different identities were presented, without repetition of the same identity in consecutive pictures. Male and female deviant faces elicited similar vMMN in both SOA conditions at around 200–500 ms latency. The results suggest that vMMN is a reliable index of regularity violations in facial gender categories.

Takács et al. (2013) investigated electrophysiological correlates of the oblique effect under unattended and attended conditions using an oddball design. The oblique effect refers to that the perceptual system is more sensitive to cardinal (vertical and horizontal) than oblique line orientations. Task-irrelevant Gábor patches elicited no vMMN when a moderate 50° change in orientation was presented. However, in the subsequent attentive condition it was found that changes as 10° in cardinal direction and 17° in oblique direction were behaviorally detectable. When 90° change was applied in the following vMMN experiment, deviations from the cardinal angels elicited larger and more sustained vMMN than those from oblique angles. Sufficiently large magnitude of change thus elicited typical oblique effect as indexed by vMMN.

Müller et al. (2013) used vMMN to study whether object formation happens in the absence of attention. Using an elegant design, participants were presented with two symmetrically arranged ellipses, and two discs of either lower or higher luminance. In separate blocks, the discs were either frequently enclosed in one ellipse or in both ellipses (standard). Occasionally, the frequent disc-to-ellipse assignment was randomly changed (deviant), allowing the investigation of ERPs to unexpected configurational changes in the arrangement of discs and ellipses. Task-irrelevant changes in disc-to-ellipse assignment resulted in increased reaction times, indicating that an unpredicted change in a task-irrelevant feature (assignment to ellipse) of the otherwise attended discs captured some of the attentional resources available for the processing of the behaviorally relevant feature (luminance). VMMN emerged in the 246–280 ms interval at posterior sites, which was localized to the inferior temporal gyrus. These results indicate that the visual system automatically registers the probability of different features to occur together in spatial proximity, i.e., to form objects.

Developmental studies on vMMN are rare. Cleary et al. (2013) investigated vMMN in 8–12 year old children and 18–42 year old adults in an oddball paradigm using low spatial frequency grating stimuli as a deviant while participants performed a centrally presented target detection task. VMMN components were observed in the 130–200 and 200–275 ms intervals in children, and in the 130–200 ms interval in the adult group at posterior electrodes. The results confirm that vMMN can be observed in 8–12 years old children and the authors suggests it as a potential tool to study visual information processing deficits in children with neurodevelopmental disabilities.

Little attention has been devoted to studying the contribution of phase reorganization vs. evoked activity, and contribution of evoked vs. induced activity to vMMN generation. Stothart and Kazanina (2013) investigated differences in phase-locking and induced activity in ERPs to rare unattended changes in spatial frequency of vertical bar stimuli in an oddball paradigm. Participants engaged in a central primary task. The results of time-frequency analyses show that vMMN—similarly to auditory MMN—is associated with an increase in phase-locking at ~100–250 ms in the theta range, which was followed by a decrease in induced power in the ~380–580 ms interval in the higher alpha range. The authors conclude that increase in theta phase-locking may reflect the higher functional coupling between cortical areas involved in the vMMN response.

Finally, in a review paper Stefanics et al. (2014) argues that the vMMN brain potential is a perceptual prediction error response, i.e., it represents the difference between the expected and the observed stimulus. Besides placing the vMMN in the hierarchical predicting coding framework, the issues of neural refractoriness, methods to control attention, and the link between veridical perception and vMMN have been discussed in the paper.

In summary, the variety of studies presented here shows that similarly to its auditory counterpart, visual MMN is a useful tool to investigate a wide range of aspects of predictive perceptual processes, including automatic stimulus discrimination, change detection, and attention-related effects. Besides indicating that vMMN has the potential to become a widely used tool in basic research, the Topic also highlighted the clinical relevance of vMMN. Regarding future directions, we believe that the field in general would greatly benefit from bridging the gap between visual MMN and research on repetition suppression or stimulus-specific adaptation, and that future vMMN studies should recognize the relevance of predictive coding theories.

## Conflict of interest statement

The authors declare that the research was conducted in the absence of any commercial or financial relationships that could be construed as a potential conflict of interest.
